# Utilisation of prehospital emergency medical services for hyperglycaemia: A community-based observational study

**DOI:** 10.1371/journal.pone.0182413

**Published:** 2017-08-03

**Authors:** Melanie Villani, Natalie Nanayakkara, Sanjeeva Ranasinha, Arul Earnest, Karen Smith, Georgia Soldatos, Helena Teede, Sophia Zoungas

**Affiliations:** 1 Monash Centre for Health Research and Implementation–MCHRI, School Public Health and Preventive Medicine, Monash University in partnership with Monash Health, Locked Bag 29, Clayton, Victoria, Australia; 2 Research and Evaluation, Ambulance Victoria, Doncaster, Victoria, Australia; 3 Diabetes and Vascular Medicine Unit, Monash Health, Clayton, Victoria, Australia; 4 Department of Epidemiology and Preventive Medicine, School of Public Health and Preventive Medicine, Monash University, Prahran, Victoria, Australia; 5 Department of Emergency Medicine, School of Primary, Aboriginal and Rural Health Care, University of Western Australia, Crawley, Western Australia, Australia; 6 The George Institute for Global Health, Camperdown, New South Wales, Australia; Catholic University, ITALY

## Abstract

**Aims:**

This study examines prehospital Emergency Medical Service (EMS) utilisation and patterns of demand for hyperglycaemia management, including characteristics of individuals and factors related to hospital transport.

**Materials and methods:**

A state-wide, community-based observational study of all patients requiring prehospital EMS for hyperglycaemia during a 7 year study period (Jan 2009–Dec 2015) using electronic data from the Ambulance Victoria data warehouse was conducted. Pre-specified variables related to patient demographics, comorbidities, examination findings, paramedic treatment and transport outcomes were obtained. Logistic regression was used to assess factors associated with transport to hospital.

**Results:**

There were 11,417 cases of hyperglycaemia attended by paramedics during the study period, accounting for 0.3–0.4% of the total annual EMS caseload, and equating to 0.54 attendances per 100 people with diabetes in the state of Victoria, Australia, per year. There was a significant increase in annual utilisation, with a rate ratio of 1.62 between 2009 (2.42 cases per 10,000 population) and 2015 (3.91 cases per 10,000 population). Fifty-one percent of cases had type 2 diabetes, 37% had type 1 diabetes, 4% had diabetes with the type unspecified and 8% had no recorded history of diabetes. Ninety percent of cases were transported to hospital. Factors associated with increased odds of transport to hospital included no known history of diabetes, regional/rural locations, case time between 0600 and <1800 hours, increasing number of comorbidities and increasingly unstable vital sign observations.

**Conclusion:**

There is substantial utilisation of prehospital EMS for hyperglycaemia. With increased population prevalence of diabetes predicted, further research on opportunities for prevention, as well as optimal management in the prehospital environment is warranted.

## Introduction

Hyperglycaemia, often in the context of uncontrolled diabetes, presents a substantial healthcare burden [1]. Hyperglycaemia increases the risk of adverse health outcomes in a range of emergency presentations including myocardial infarction [2], stroke [3] [4], sepsis [5] and trauma [6] and is a strong predictor of increased length of hospital stay and in-hospital mortality [7]. At the extreme, Diabetic Ketoacidosis (DKA) and Hyperglycaemic Hyperosmolar State (HHS) are potentially fatal. The annual hospital discharge rate for DKA has been reported as 22.0 per 1,000 population with diabetes in the US [8] and the incidence of intensive care unit (ICU) admissions for DKA is increasing [9]. Mortality of HHS (between 5 and 20% [10] [11]) is higher than that of DKA (< 1%–4% [11] [12] [13]), attributed to dehydration, age and the presence of comorbidities [14]. Acute hyperglycaemic crises, including HHS and DKA, are potentially preventable with education, health care provider communication during illness and better access to medical care [15]. Given the potential for prevention, the morbidity and mortality associated with hyperglycaemia [16], and the increasingly significant health and financial burden of diabetes, a greater understanding of current prehospital Emergency Medical Service (EMS) use for hyperglycaemia may inform interventions to improve future utilisation. This Australian state-wide, community-based study examines 1) utilisation patterns and temporal trends of demand for prehospital EMS for hyperglycaemia in Victoria, 2) characteristics of, and patterns of care for individuals receiving EMS assistance for hyperglycaemia and 3) factors associated with transport to hospital following attendance by EMS for hyperglycaemia.

## Materials and methods

### Study design

This 7 year, retrospective observational study was conducted on all cases of hyperglycaemia attended by Ambulance Victoria (AV) between 01/01/2009 and 31/12/2015. During this time approximately 3 months of data (26/09/2014–20/12/2014) was unavailable due to lapse in electronic data collection linked to industrial action. Individuals of all ages receiving prehospital emergency medical assistance from AV during the study period, with a documented primary assessment of “hyperglycaemia” and an initial blood glucose level (BGL) greater than 7.8mmol/l were included. The primary assessment, assigned by the attending paramedic, is the main presenting problem at the time the patient is discharged from ambulance care. A BGL threshold of 7.8 mmol/L was selected as per the ADA (American Diabetes Association) definition of hyperglycaemia in hospitalised patients [17]. Monash Health Human Research Ethics committee approved this study.

### Setting

The state of Victoria, Australia has with an estimated population of 5.94 million in 2015 [18], 300,960 of whom were registered as having diabetes [19]. Victoria is serviced by a two-tiered prehospital EMS system, AV, which responds with Advanced Life Support (ALS) or Intensive Care (MICA) paramedics to approximately 550,000 emergency cases annually [20]. Every case attended by AV is recorded by the attending paramedic, using the VACIS®, an electronic patient care record and integrated data warehouse [21]. In this study de-identified data was used with no ability to distinguish repeat callers, thus repeat attendances are treated as individual cases.

### Variables

Pre-specified, de-identified data related to EMS operational processes (geographic location, dispatch urgency, time of request) and patient characteristics (type of diabetes, gender, age, scene type, medical history, prescribed medications, examination findings, treatments administered and EMS transport outcomes) were obtained from the AV data system. Investigators had full access to the de-identified data following extraction.

### Operational process variables

The location of the EMS attendance was classified as “metropolitan” or “regional/rural” according to the Australian Department of Immigration postcode classification [22]. At the point of call, urgency categories are assigned as per the Medical Priority Dispatch System [23] [24] and used to determine the type of response received; code 1 (lights and sirens response), code 2 (acute but not time critical response) and code 3 (non-urgent, routine response). In the current study, the time of the emergency call request was grouped into four 6 hour time periods (2400 to <0600 hours, 0600 to <1200 hours, 1200 to <1800 and 1800 hours to < 2400 hours).

### Patient-related variables

Diabetes type, based on patient/bystander self-report was classified as type 1 diabetes; type 2 diabetes; unspecified diabetes type or no history of diabetes. Scene type was classified by paramedics as: private residence, residential care facility/supported accommodation, general practitioner (GP) clinic, hospital, public place or other. Medical history, also based on self- or family-reports, is recorded by paramedics at the time of care and does not follow a standardised approach. A selection of comorbidities considered to be commonly associated with diabetes and/or utilisation of prehospital emergency services were examined; myocardial infarction, ischaemic heart disease, hypertension, hypercholesterolemia, stroke, anxiety, depression, renal impairment and concurrent infection. Initial examination findings as recorded by the paramedics included Glasgow Coma Score (GCS), blood glucose level (BGL, mmol/L), systolic blood pressure (BP, mmHg), heart rate (beats/min) and respiratory rate (breaths/min). Glucometry is performed by capillary blood samples and point of care glucometers (Freestyle Optium®, Abbott Laboratories, England) with a valid range of 1.1 mmol/l to 27.8 mmol/l. Values below and above this range are displayed and recorded as “low” or “high” respectively. Where BGL readings of “high” were obtained, data was re-coded to 27.9mmol/L for the purpose of analysis. Treatments administered for hyperglycaemia were varied and administration of normal saline, anti-emetics, oxygen, cardiac and analgesic therapy were examined. Ketone assessment and the administration of insulin are not included in the scope of practice of ALS and MICA road paramedics in AV.

### Statistical analysis

The annual number of cases of hyperglycaemia attended by AV were tabulated and seasonal distribution patterns were examined. Annual event rates (per 10,000) were calculated by dividing the annual caseload by total resident population for each year as well as by population registered as having diabetes (per 100) and Poisson regression analysis was used to calculate the rate ratio of the initial and final study year. Metropolitan and regional/rural event rates were calculated by dividing the annual caseload by residential population separately for metropolitan and regional/rural locations. At the final year of data collection, Victoria had an estimated resident population of 5.94 million, consisting of 4.53 million in Greater Metropolitan Melbourne and 1.41 million in regional/rural Victoria [18] with 300,960 registered as having diabetes [19]. Categorical variables were reported as percentages and differences between subgroups analysed using χ2 test. Continuous variables were summarised as means with standard deviations or as medians with interquartile ranges and subgroup analysis performed by analysis of variance (ANOVA), Mann-Whitney U or Kruskal Wallis test as appropriate. No imputation of missing data was performed as the proportion of missing data across any variable was less than 3.5%.

To examine factors associated with transport to hospital by EMS, logistic regression models were used overall in a 2-step approach. In the first step, crude models were calculated. The selection of variables was based on identifying all measured clinical variables of known or suspected importance for decision to transport. In the second step, models were adjusted for all covariates identified to yield a p-value < 0.05 in the univariable analysis (diabetes type, age, geographic location, case time, myocardial infarction, stroke, infection, renal impairment, number of comorbidities, sulphonylurea, biguanide, dispatch code, initial blood glucose level (BGL), initial blood pressure (BP), initial heart rate (HR), initial Glasgow Coma Score (GCS) and initial respiratory rate (RR)). A two-sided significance level of 0.05 was considered statistically significant for all hypothesis tests. All analyses were performed using Stata software version 12.0 (StataCorp, Texas, USA).

## Results

### Overall demand

**Fig 1:**
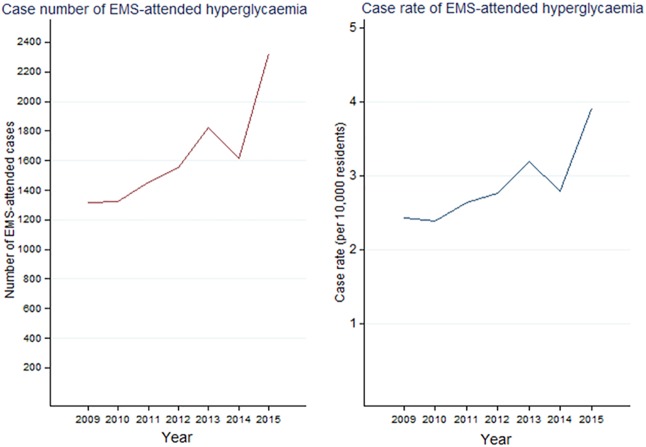
Annual case number and annual case rate of EMS-attended hyperglycaemia. During 2014, 3 months of data was unavailable due to lapse in electronic data collection.

The total number of attendances during the study period was 11, 417 cases, equating to 0.54 attendances per 100 people with diabetes in Victoria, per year and 2.87 attendances per 10,000 Victorian residents per year. There was an increase in annual case rate from 2.42 to 3.91 cases per 10,000 Victorian residents between 2009 and 2015 (Fig 1), generating a rate ratio of 1.62 [95% CI: 1.51, 1.73] (p<0.001) between 2009 and 2015. The average annual rate of attendance over the study period was higher for regional/rural locations (3.17 per 10,000 per year) compared to metropolitan locations (2.77 per 10,000 residents, per year). A modest seasonal trend was observed, with a greater proportion of cases in winter (26.5%) compared to spring (23.2%).

### Patient characteristics

**Fig 2:**
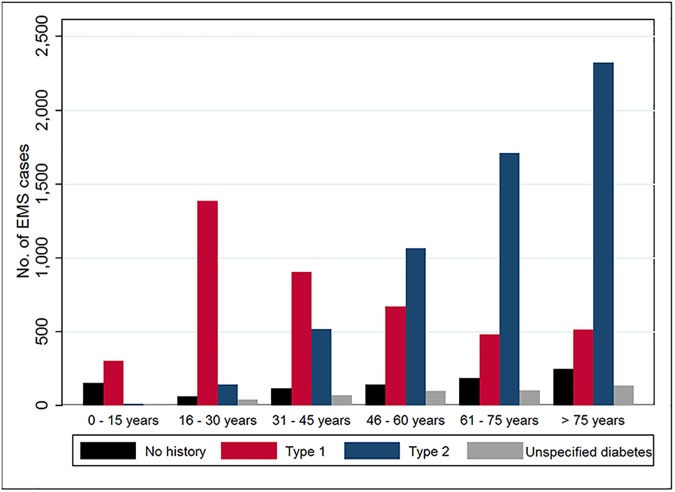
EMS attendances by age group and diabetes type.

Patient characteristics are reported in Table 1. Of people using EMS for hyperglycaemia, the largest proportion was from people with type 2 diabetes (50.6%), followed by type 1 (37.4%), 4.0% had unspecified diabetes type and 8.0% had no history of diabetes. The mean age (±SD) was 56.8 ± 23.6 years, and attendances to people with type 1 diabetes were younger on average (42.2 ± 22.5 years) than to those with type 2 diabetes (68.0 ± 16.8 years). Cases with type 1 diabetes were greatest in the 16 to 30 year age group and declined with advancing age whereas cases with type 2 diabetes increased with advancing age (Fig 2). Equal numbers of males and females were attended. The majority of attendances were between midday and 1800 hours (31.1%) and the minority between midnight and 0600 hours (14.2%). Private residences (64.8%) were the most common scene type however 14.7% of cases were to residential care facilities/supported accommodation. Comorbid conditions were recorded in 84% of cases, comprising hypertension in over a third of cases, hypercholesterolaemia in over a quarter of cases and depression in 15% of cases. Prescribed patient medications included (not limited to) insulin (68.6%), biguanide (metformin) (23.5%) and sulphonylurea (15.7%).

**Table 1:** Characteristics of patients who utilise EMS for hyperglycaemia.

	*All Cases*	*Diabetes Type*			
				Unspecified	No
		Type 1	Type 2	diabetes	diagnosis of
	1417	n = 4266	n = 5775	type	diabetes
	(100%)	(37.4%)	(50.6%)	n = 461	n = 915
				(4.0%)	(8.0%)
***Gender***	N (%)	N (%)	N (%)	N (%)	N (%)
*Male*	5,702 (50.0)	2,136 (50.1)	2,793 (48.4)	243 (52.7)	530 (58.0)
*Female*	5,710 (50.0)	2,130 (49.9)	2,978 (51.6)	218 (47.3)	384 (42.0)
***Age***					
*Age (mean ± SD)*	56.8 ± 23.6	42.2 ± 22.5	68.0 ± 16.8	59.5 ± 21.8	53.4 ± 27.6
***Geographic Location***					
*Metro*	8,301 (72.7)	2,923 (68.5)	4,468 (77.4)	283 (61.4)	627 (68.5)
*Regional/Rural*	3,116 (27.3)	1,343 (31.5)	1,307 (22.6)	178 (38.6)	288 (31.5)
***Case Time***					
*2400 to <0600*	1,625 (14.2)	715 (16.8)	750 (13.0)	65 (14.1)	95 (10.4)
*0600 to <1200*	3,389 (29.7)	1,340 (31.4)	1,641 (28.4)	115 (25.0)	293 (32.1)
*1200 to <1800*	3,548 (31.1)	1,197 (28.1)	1,877 (33.5)	147 (31.9)	327 (35.4)
*1800 to <2400*	2,850 (25.0)	1014 (23.8)	1,504 (26.1)	134 (29.1)	198 (21.7)
***Scene Type***					
*Private residence*	7,401 (64.8)	2,842 (66.6)	3,820 (66.2)	310 (67.3)	429 (46.9)
*Residential Care*	1,678 (14.7)	420 (9.9)	1,066 (18.5)	58 (12.6)	134 (14.6)
*General practitioner clinic*	564 (4.9)	169 (4.0)	273 (4.7)	18 (3.9)	104 (11.4)
*Hospital*	625 (5.5)	302 (7.1)	160 (2.8)	25 (5.4)	138 (15.1)
*Public place*	728 (6.4)	313 (7.3)	312 (5.4)	37 (8.0)	66 (7.2)
*Other*	421 (3.7)	220 (5.2)	144 (2.5)	13 (2.8)	44 (4.8)
***Comorbidities***					
*Myocardial infarction*	703 (6.2)	161 (3.8)	481 (8.3)	28 (6.1)	33 (3.6)
*Ischaemic heart disease*	1,133 (9.9)	253 (5.9)	776 (13.4)	50 (10.9)	54 (5.9)
*Hypertension*	4,282 (37.5)	886 (20.8)	2,994 (51.8)	152 (33.0)	250 (27.3)
*Hypercholesterolemia*	2,924 (25.6)	639 (15.0)	2,047 (35.5)	101 (21.9)	137 (15.0)
*Stroke*	1,020 (8.9)	201 (4.7)	697 (12.1)	49 (10.6)	73 (8.0)
*Infection*	1,195 (10.5)	282 (6.6)	753 (13.0)	48 (10.4)	112 (12.2)
*Anxiety*	545 (4.8)	180 (4.2)	295 (5.1)	23 (5.0)	47 (5.1)
*Depression*	1,729 (15.1)	614 (14.4)	936 (16.2)	62 (13.5)	117 (12.8)
*Renal impairment*	665 (5.8)	225 (5.3)	395 (6.8)	26 (5.6)	19 (2.1)
***Number of comorbidities***					
*0 comorbidities*	1,780 (15.6)	1,006 (23.6)	271 (4.7)	125 (27.1)	378 (41.3)
*1 comorbidity*	1,761 (15.7)	922 (21.6)	648 (11.2)	63 (13.7)	128 (13.9)
*2 comorbidities*	1,836 (16.3)	724 (17.0)	940 (16.3)	70 (15.2)	102 (11.2)
*3 comorbidities*	1,652 (14.7)	526 (12.3)	959 (16.6)	68 (14.8)	99 (10.8)
*≥ 4 comorbidities*	4,388 (39.0)	1,088 (25.5)	2,957 (51.2)	135 (29.3)	208 (22.7)
***Medications***					
*Sulphonylurea*	1,797 (15.7)	120 (2.81)	1,597 (27.7)	80 (17.4)	0
*Biguanide*	2,678 (23.5)	254 (6.0)	2,296 (39.8)	128 (27.8)	0
*Insulin*	7,831 (68.6)	4,266 (100.0)	3,381 (58.6)	184 (39.9)	0

### Operational, assessment and treatment characteristics

Operational, assessment and treatment characteristics are reported in Table 2. Over half of all cases of hyperglycaemia were classified code 1 (“lights and sirens”) response. Patients with type 1 diabetes had a higher proportion of code 1 responses (56.3%) than those with type 2 diabetes (49.5%). At initial paramedic assessment the median [IQR] BGL was 24.5 [19.8, 27.9] mmol/L and 36% of patients had an initial BGL reading >27.8mmol/L. The median [IQR] GCS was 15 [14, 15] and respiratory rate was 18 [16, 20] breaths/min. The mean (±SD) systolic blood pressure was 129.3 ± 26.3 mmHg and heart rate was 94.3 ± 21.0 beats/min. Treatments administered by paramedics included normal saline (21.5%), oxygen therapy (19.8%), anti-emetics (3.2%), analgesia (morphine, fentanyl and methoxyflurane, each administered in 1–2% of cases) and aspirin, glyceryl trinitrate and midazolam (all <1.0%).

**Table 2:** EMS operational, assessment and treatment characteristics.

	*All Cases*	*Diabetes Type*			
				Unspecified	No history
		Type 1	Type 2	diabetes	of diabetes
	n = 11417			type	
	(100%)	n = 4266	n = 5775	n = 461	n = 915
		(37.4%)	(50.6%)	(4.0%)	(8.0%)
***Dispatch Code***					
*Code 1 (Lights and sirens)*	5,914 (51.8)	2,401 (56.3)	2,860 (49.5)	239 (51.8)	414 (45.3)
*Code 2 (acute)*	4,600 (40.3)	1,629 (38.2)	2,418 (41.9)	180 (39.1)	373 (40.8)
*Code 3 (non-urgent)*	903 (7.9)	236 (5.5)	497(8.6)	42 (9.1)	128 (14.0)
***Initial Examination***					
*BGL (mmol/L) (median [IQR])*	24.5 [19.8, 27.9]	25.5 [20.2, 27.9]	23.6 [19.5, 27.9]	25.3 [19.9, 27.9]	26.0 [19.8, 27.9]
*Systolic Blood Pressure (mmHg) (mean ± sd)*	129.3 ± 26.3	124.0 ± 25.5	133.7 ± 26.3	131.3 ± 25.1	125.1 ± 26.0
*Heart rate (bpm) (mean ± sd)*	94.3 ± 21.0	99.3 ± 21.1	90.5 ± 19.7	91.9 ± 20.4	97.1 ± 23.4
*GCS (median [IQR])*	15 [14, 15]	15 [15, 15]	15 [14, 15]	15 [15, 15]	15 [14, 15]
*Resp. Rate (bpm) (median [IQR])*	18 [16, 20]	18 [16, 24]	18 [16, 20]	18 [16, 20]	18 [16, 24]
***Paramedic Treatment***					
*Normal saline*	2,457 (21.5)	1,055 (24.7)	1,126 (19.5)	73 (15.8)	203 (22.2)
*Anti-emetic*	361 (3.2)	231 (5.4)	110 (1.9)	11 (2.4)	9 (1.0)
*Oxygen therapy*	2,214 (19.8)	798 (19.5)	1,102 (19.4)	93 (21.0)	221 (24.8)
*Aspirin*	101 (0.9)	36 (0.8)	57 (1.0)	2 (0.4)	6 (0.7)
*Glyceryl trinitrate*	58 (0.5)	20 (0.5)	31 (0.5)	1 (0.2)	6 (0.7)
*Morphine*	203 (1.8)	107 (2.5)	76 (1.3)	6 (1.3)	14 (1.5)
*Fentanyl*	144 (1.3)	89 (2.1)	45 (0.8)	1 (0.2)	9 (1.0)
*Methoxyflurane*	213 (1.9)	130 (3.1)	66 (1.1)	4 (0.9)	13 (1.4)
*Midazolam*	10 (0.1)	5 (0.1)	4 (0.1)	0 (0.0)	1 (0.1)

### Transport to hospital

Overall, 90.1% of cases attended by EMS for hyperglycaemia were transported to hospital. When patients were not transported to hospital with EMS, documented reasons included patient refusing transport (40.5%), paramedics deeming transport not required (30.2%), patient to follow up with local medical officer (12.7%), patient transported by private means (9.5%) or other reason (7.2%).

The proportions transported and odds ratios of associated factors are reported in Table 3. Factors related to increased likelihood of hospital transport in multivariable analysis included no diagnosed history of diabetes (AOR 2.86 [1.74, 4.69] p<0.001) (reference: unspecified diabetes type), case in a regional/rural location (AOR 1.51 [1.27, 1.78] p<0.001) (reference: metropolitan location), case time 0600 to <1200 hours (AOR 1.55 [1.24, 1.93] p<0.001) and 1200 to <1800 hours (AOR 1.54 [1.24, 1.91] p<0.001) (reference case time: 2400 to < 0600) and dispatch code 3 (1.89 [1.38, 2.59] p<0.001 (reference: dispatch code 1). Compared to those with no comorbidities, the likelihood of transport to hospital increased with the number of recorded comorbidities, from 2 (AOR 1.65 [1.28, 2.14] p<0.001) to 4 or more comorbidities (AOR 2.49 [1.93, 3.22] p<0.001). At initial assessment, increased BGL, heart rate and respiratory rate were associated with increased odds of transport. An increase in BGL of 10 mmol/L, an increase in heart rate of 10 beats/min and an increase in respiratory rate of 4 breaths/min were associated with a 153%, 27% and 57% increase in odds of transport to hospital respectively (Table 3). Blood pressure was not significantly associated with odds of hospital transport. In contrast, a higher GCS at initial assessment was associated with reduced likelihood of transport to hospital.

**Table 3:** Transport frequency and unadjusted and adjusted odds of transport to hospital.

Characteristic (% transported)	OR [95% CI]	AOR [95% CI]
**Diabetes Type**		
Unspecified (87.4%)	[Reference]	
Type 1 (91.6%)	1.57 [1.17, 2.11]*	1.37 [0.97, 1.95]
Type 2 (88.3%)	1.09 [0.82, 1.45]	1.02 [0.73, 1.43]
Undiagnosed (95.3%)	2.92 [1.93, 4.41]*	2.86 [1.74, 4.69]*
**Gender**		
Male (89.8%)	[Reference]	
Female (90.4%)	1.07 [0.95, 1.20]	
**Age Category (years)**		
0–15 (94.1%)	[Reference]	
16–30 (92.8%)	0.81 [0.53, 1.23]	1.13 [0.67, 1.98]
31–45 (90.1)	0.57 [0.37, 0.86]*	0.76 [0.46, 1.28]
46–60 (87.8%)	0.45 [0.30, 0.67]*	0.62 [0.37, 1.03]
61–75 (89.0%)	0.50 [0.34, 0.75]*	0.65 [0.38, 1.09]
>75 (90.3%)	0.58 [0.39, 0.86]*	0.68 [0.40, 1.16]
**Geographic Location**		
Metro (89.4%)	[Reference]	
Regional (91.8%)	1.33 [1.15, 1.54]*	1.51 [1.27, 1.78]*
**Case Time**		
2400 to <0600 (86.5%)	[Reference]	
0600 to <1200 (93.1%)	1.52 [1.25, 1.85]*	1.55 [1.24, 1.93]*
1200 to <1800 (91.6%)	1.42 [1.17, 1.72]*	1.54 [1.24, 1.91]*
1800 to <2400 (86.5%)	0.84 [0.70, 1.01]	0.92 [0.74, 1.13]
**Comorbidities**		
Myocardial infarction (92.5%)	1.38 [1.03, 1.84]*	1.45 [1.05, 2.00]*
Ischaemic heart disease (91.1%)	1.14 [0.92, 1.41]	-
Hypertension (89.7%)	0.94 [0.83, 1.07]	-
Hypercholesterolaemia (89.5%)	0.92 [0.80, 1.05]	-
Stroke (93.8%)	1.74 [1.34, 2.27]*	1.39 [1.04, 1.86]*
Infection (93.9%)	1.78 [1.39, 2.28]*	1.31 [0.99, 1.72]
Anxiety (89.5%)	0.94 [0.71, 1.25]	-
Depression (90.9%)	1.12 [0.93, 1.33]	-
Renal impairment (92.6%)	1.41 [1.04, 1.90]*	1.16 [0.83, 1.61]
**No. of Comorbidities.**		
0 (87.4%)	[Reference]	
1 (87.6%)	1.02 [0.83, 1.25]	1.28 [0.99, 1.64]
2 (89.2%)	1.19 [0.96, 1.46]	1.65 [1.28, 2.14]*
3 (89.2%)	1.19 [0.96, 1.48]	1.75 [1.34, 2.28]*
≥4 (92.4%)	1.76 [1.47, 2.12]*	2.49 [1.93, 3.22]*
**Medications**		
Sulphonylurea (88.5%)	0.83 [0.70, 0.97]*	1.03 [0.85, 1.24]
Biguanide (87.2%)	0.68 [0.59, 0.78]*	0.97 [0.77, 1.16]
Insulin (90.0%)	0.98 [0.86, 1.12]	-
**Dispatch Code**		
Code 1 (90.4%)	[Reference]	
Code 2 (89.0%)	0.86 [0.76, 0.98]	0.98 [0.85, 1.13]
Code 3 (93.4%)	1.53 [1.16, 2.01]*	1.89 [1.38, 2.59]*
**Initial Examination**		
Initial BGL (↑10 mmol/L)	2.94 [2.60, 3.33]*	2.53 [2.21, 2.90]*
Initial BP (↑10mmHg)	0.96 [0.94, 0.98]*	1.01 [0.98, 1.04]
Initial HR (↑10bpm)	1.34 [1.29, 1.39]*	1.27 [1.21, 1.33]*
Initial GCS	0.59 [0.52, 0.66]*	0.46 [0.38, 0.55]*
Initial RR(↑4 bpm)	1.80 [1.67, 1.94]*	1.57 [1.43, 1.71]*

Adjusted Odds Ratio adjusted for diabetes type, age, geographic location, case time, myocardial infarction, stroke, infection, renal impairment, number of comorbidities, sulphonylurea, biguanide, dispatch code, initial blood glucose level (BGL), initial blood pressure (BP), initial heart rate (HR), initial Glasgow Coma Score (GCS), initial respiratory rate (RR). Results reaching statistical significance with p<0.05 are indicated with *

## Discussion

With over 11,000 cases over a 7 year period, this is the largest study to examine demand patterns, patient characteristics, prehospital management and transport outcomes of prehospital EMS utilisation for hyperglycaemia. We found a considerable increase in the annual number of cases attended throughout the study period. While the majority of attendances were for middle-age people with type 2 diabetes, requesting EMS from their private residence, 8% of cases had no history of diabetes and 15% of attendances were to residential care facilities. The overall transport frequency was high (90.1%), with multiple factors related to increased likelihood of transport No consistent approach to paramedic management of hyperglycaemia was observed.

Overall, prehospital EMS attendances for hyperglycaemia were less frequent than for hypoglycaemia (approximately 4000 cases annually [25]) however, the frequency of transport to hospital for hyperglycaemia was higher than for other conditions (approximately 80% overall in AV [26]) and much higher than for hypoglycaemia (40% [25]). This highlights the opportunity for interventions to reduce the burden to prehospital as well as hospital EMS of attendances and transport for management of hyperglycaemia.

Residential care facility patients accounted for 15% of the prehospital EMS caseload. With a high prevalence of diabetes in residential-care facility residents [27], this medically complex cohort utilise significant healthcare resources. Our study shows that this use extends to the prehospital emergency medical service system. Consistent with this, we observed an increase in utilisation with advancing age and greater likelihood of hospital transport with increasing number of comorbidities. Given that up to 31% of transfers from residential aged care facilities to hospitals are reported as potentially avoidable [28], further research is required to ascertain the proportion of residential care requests that represent acute hyperglycaemic emergencies requiring transfer as distinct from uncomplicated hyperglycaemia that could potentially be managed on site.

Eight percent of the cases in this study did not have a prior history of diabetes documented, possibly presenting with a diabetic emergency for the first time. As we were unable to distinguish between uncomplicated hyperglycaemia and the acute glycaemic emergencies (DKA and HHS), this group may have included people with reduced insulin sensitivity or ‘pre-diabetes’ as well as those with first presentation DKA or HHS. Previous studies have shown newly diagnosed diabetes accounts for 17% of hospital admission for hyperglycaemic diabetic emergencies [13], and is as high as 30% in young populations with type 1 diabetes [29]. Greater awareness among the community and primary care providers of how to optimize screening and diagnosis and avoid life threatening hyperglycaemic presentations is needed. In addition, a high proportion of cases had type 1 diabetes and were in the 16–30 year age group, suggesting a considerable number attendances for possible DKA. A focussed effort on education, phone counselling or early use of diabetes drop-in centers may assist to avert a portion of these attendances.

The current study observed no consistent approach to the management of hyperglycaemia. With no ability to test for ketones, DKA could not be detected prior to arrival at hospital and lack of evidence-based guidelines for the management of prehospital hyperglycaemia may be limiting treatment options for paramedics. This study found that although a significant number (36%) of patients had a BGL reading >27.8mmol/L, the proportion with DKA or HHS was unknown. The addition of ketone assessment to routine assessment could be used to guide treatment pathways for paramedics, potentially prompting phone consultation or expediting management after transport to hospital.

This study is strengthened by capturing all cases of hyperglycaemia attended by prehospital emergency medical services at a state-population level and could be generalisable to community populations with similar prehospital health care models. Retrospective data, with the inability to verify the data entered by the paramedics (based on patient/bystander report) and inability to distinguish repeat attendances to the same patient (due to de-identified data) were limitations. Access to age-stratified data of the Victorian diabetic population for the study period was not available, thus precluding an age-adjusted rate ratio calculation. However, it is thought that the unadjusted and age-adjusted rate ratio would not differ substantially given the demographic changes during the study period were minimal [30]. In addition, DKA cases were not able to be distinguished from uncomplicated hyperglycaemia as ketone testing was not part of routine assessment. The use of a primary assessment of hyperglycaemia as the inclusion criteria resulted in patients with “more serious” medical assessments, in addition to hyperglycaemia, being excluded from the study, thus possibly under-estimating the true incidence. The cause of the EMS request for hyperglycaemia was not captured and further research on reasons for EMS requests could help inform the proportion of preventable cases.

In conclusion, this study demonstrates the considerable EMS utilisation for hyperglycaemia, common across both metropolitan and regional/remote areas. The proportion of cases with undiagnosed diabetes utilising prehospital EMS demonstrate the need for increased awareness and screening for diabetes. Attendances to residential care facilities highlight the need for further research to determine whether interventions within facilities could avert any utilisation. Finally, guidelines or clinical pathways to support prehospital EMS management may expedite appropriate care for hyperglycaemia.

## Supporting information

S1 File Supporting dataset. (CSV)
